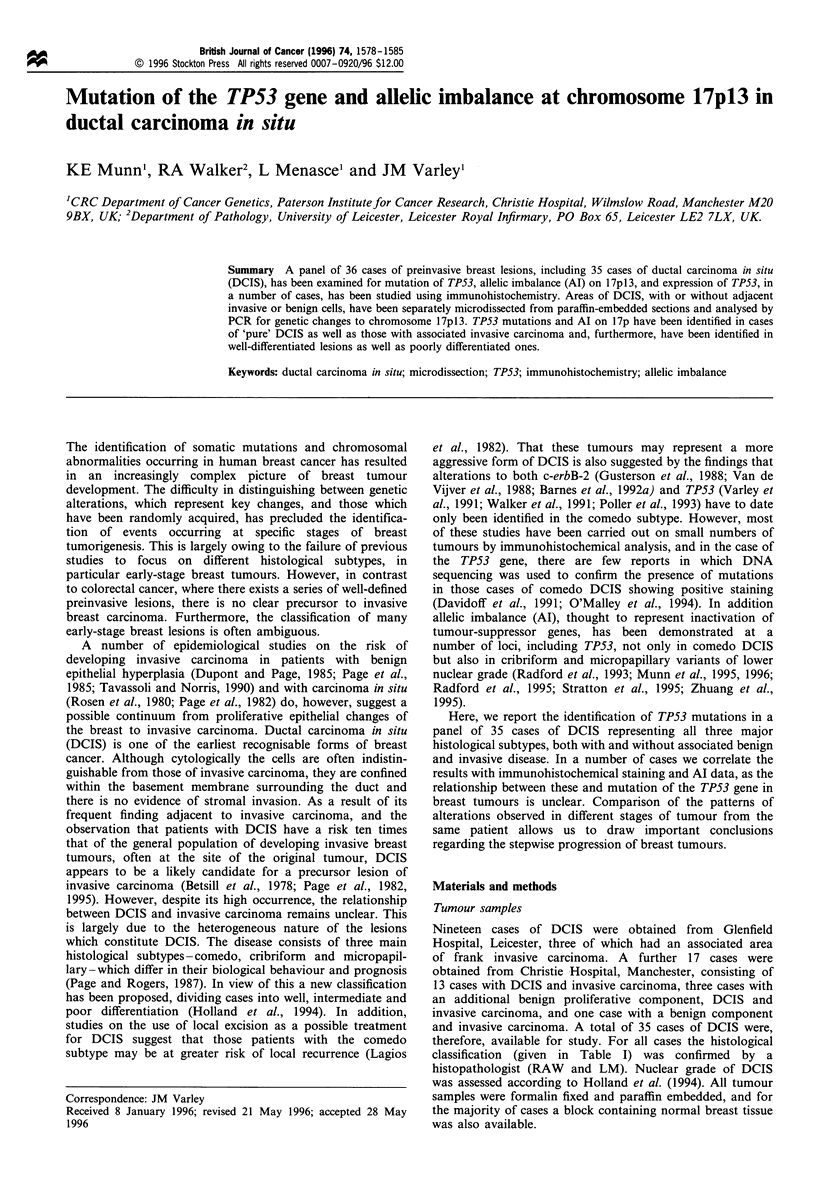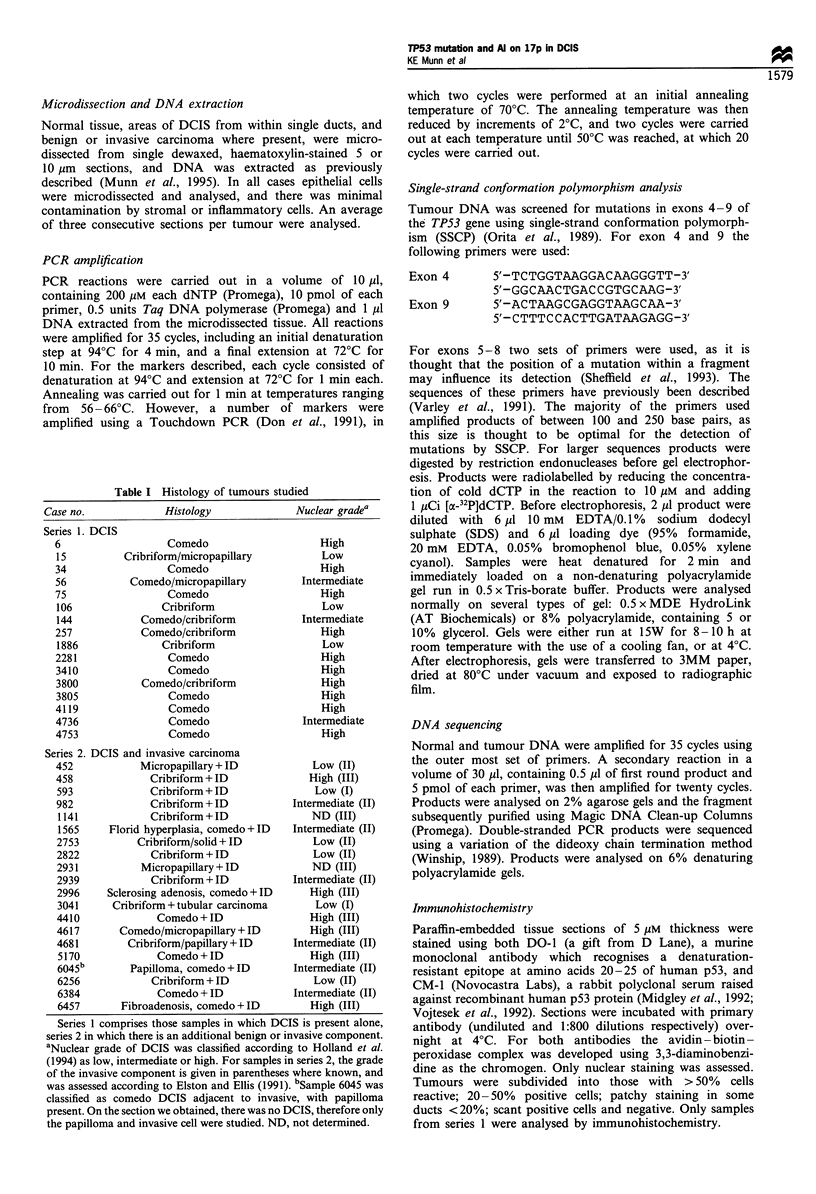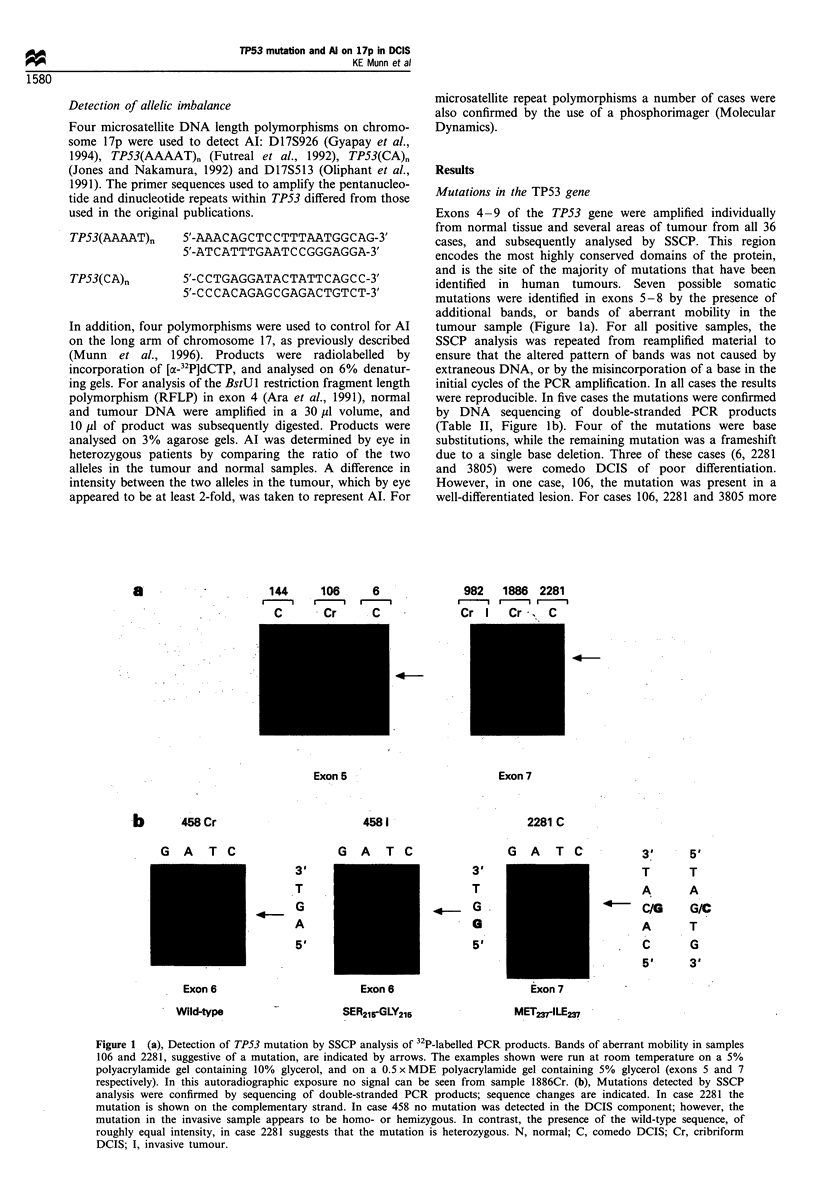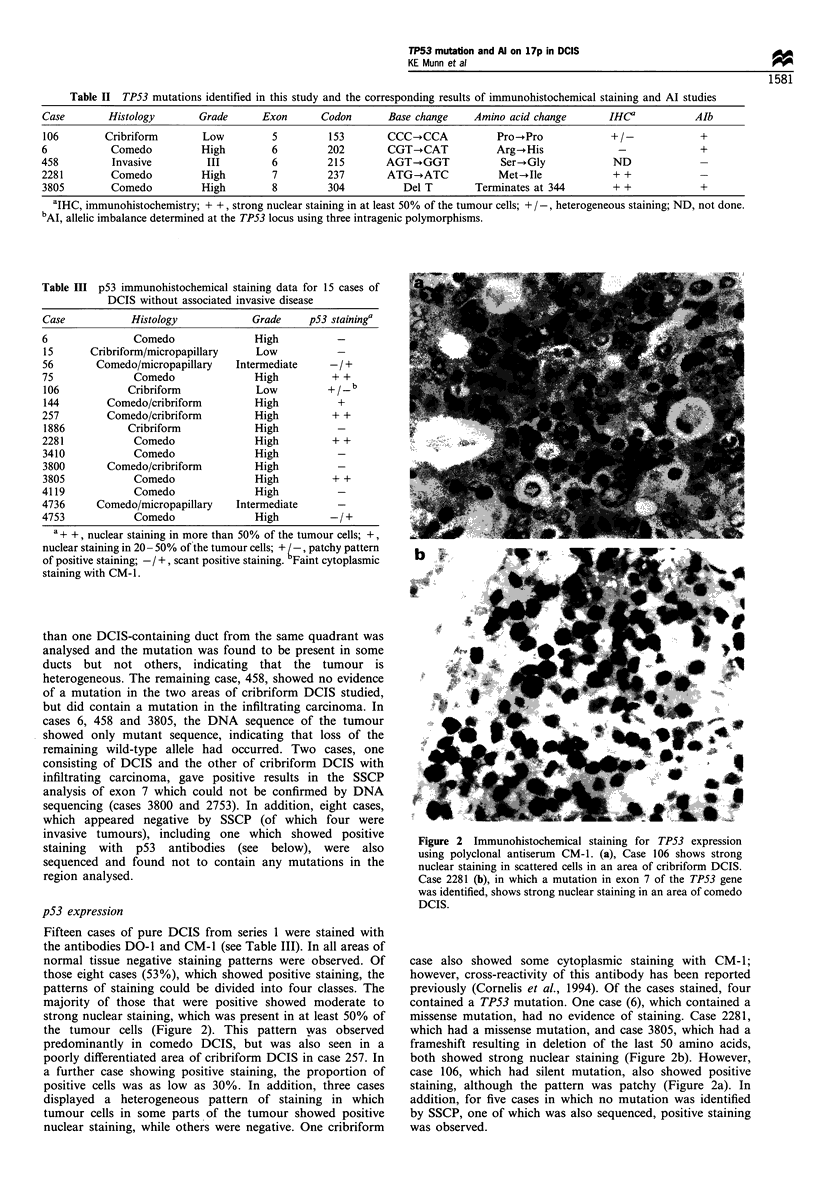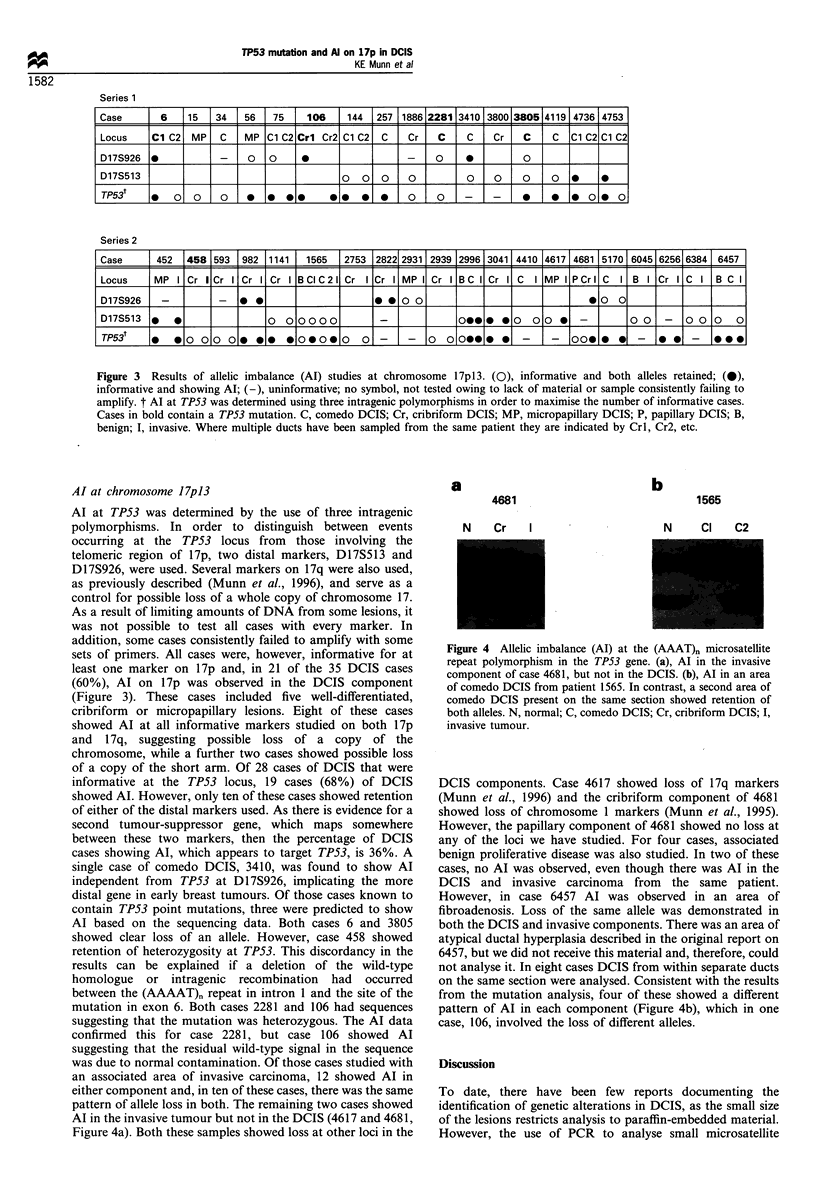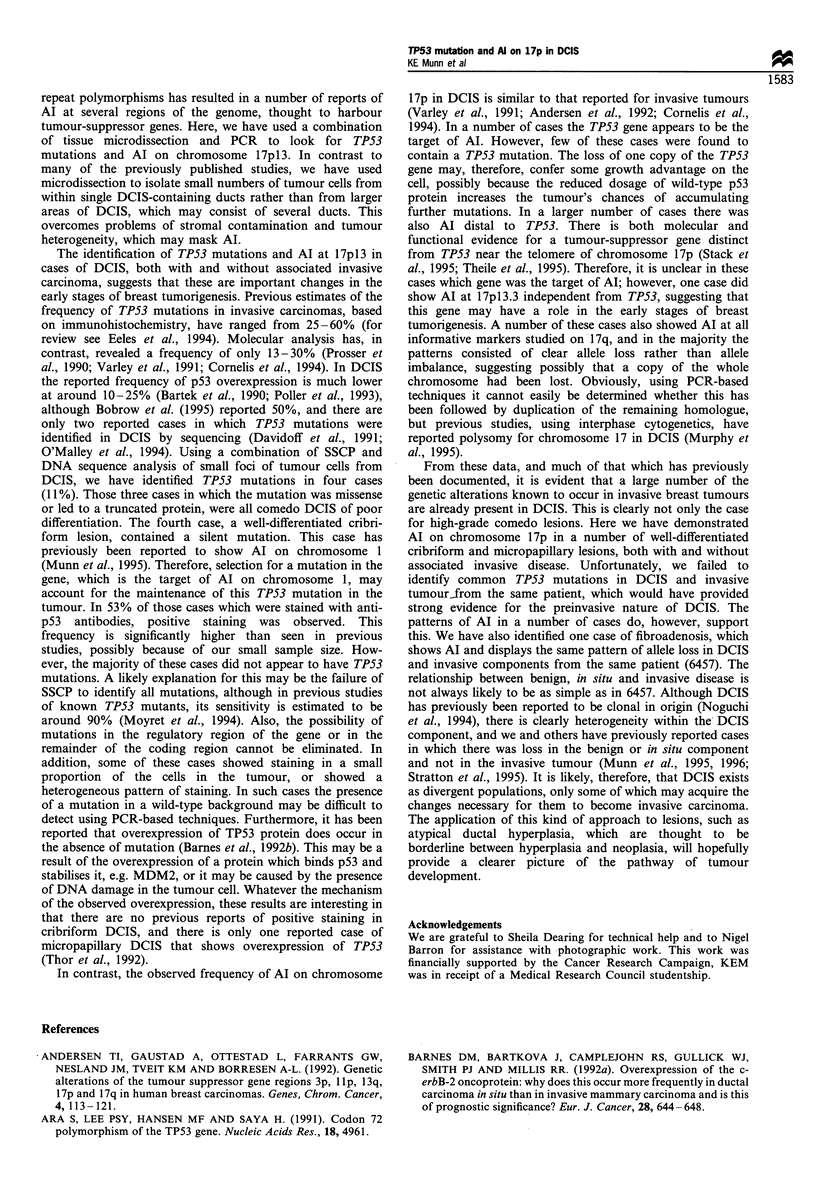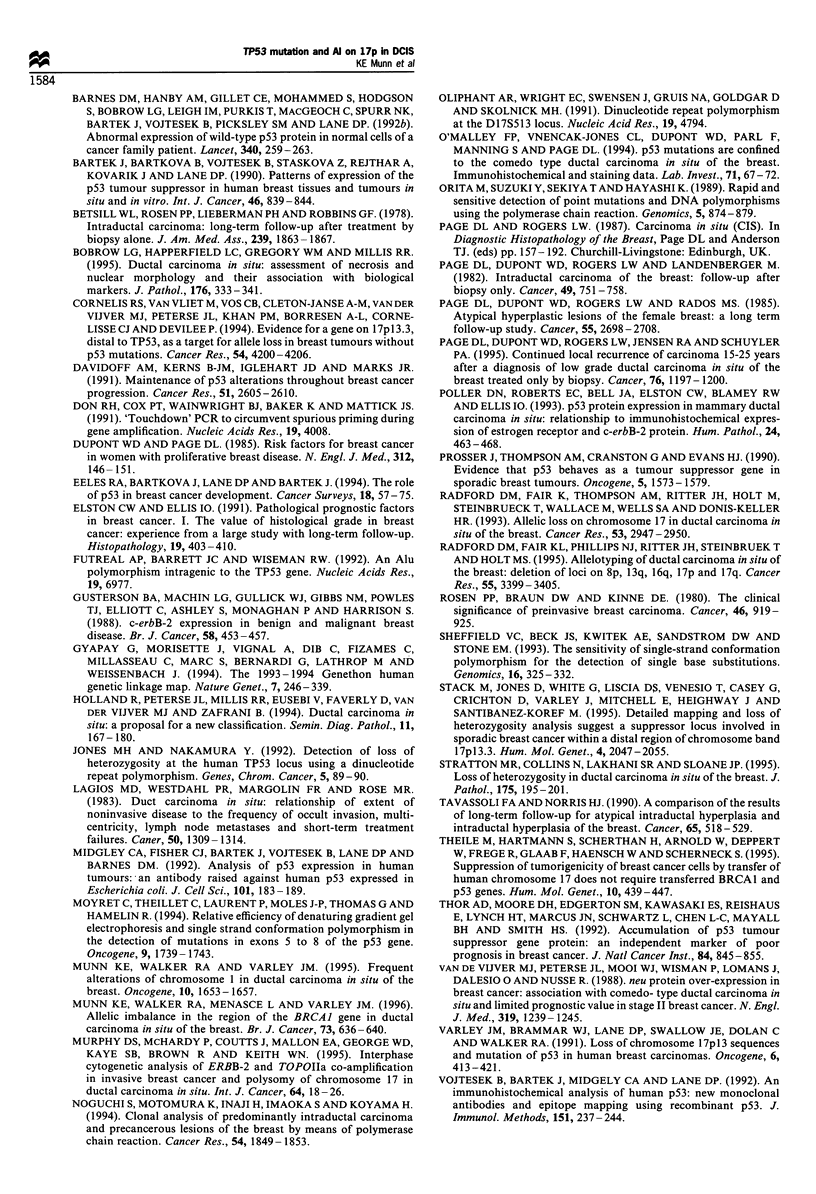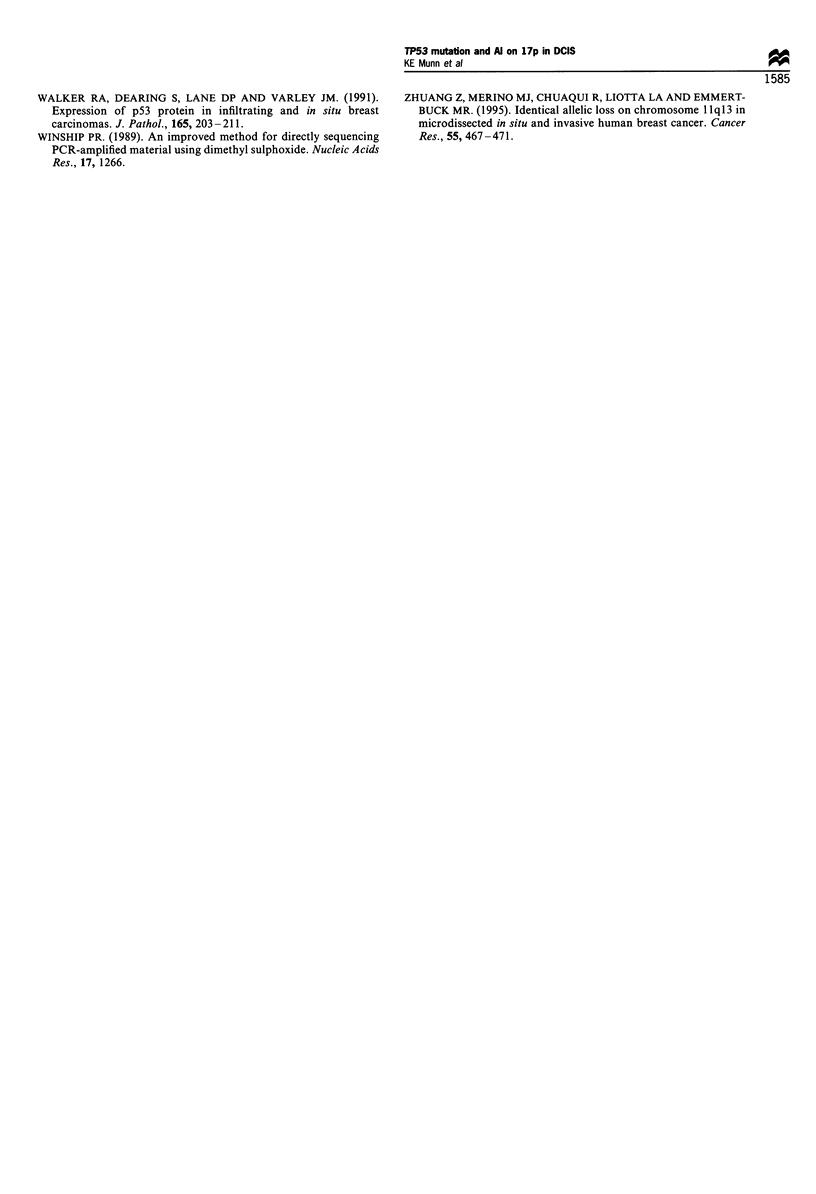# Mutation of the TP53 gene and allelic imbalance at chromosome 17p13 in ductal carcinoma in situ.

**DOI:** 10.1038/bjc.1996.592

**Published:** 1996-11

**Authors:** K. E. Munn, R. A. Walker, L. Menasce, J. M. Varley

**Affiliations:** CRC Department of Cancer Genetics, Paterson Institute for Cancer Research, Christie Hospital, Manchester, UK.

## Abstract

**Images:**


British Journal of Cancer (1996) 74, 1578–1585
© 1996 Stockton Press All rights reserved 0007–0920/96 $12.00

### Mutation of the *TP53* gene and allelic imbalance at chromosome 17p13 in ductal carcinoma *in situ*

KE Munn[1], RA Walker[2], L Menasce[1] and JM Varley[1]

[1]*CRC Department of Cancer Genetics, Paterson Institute for Cancer Research, Christie Hospital, Wilmslow Road, Manchester M20 9BX, UK;* [2]*Department of Pathology, University of Leicester, Leicester Royal Infirmary, PO Box 65, Leicester LE2 7LX, UK.*

**Summary** A panel of 36 cases of preinvasive breast lesions, including 35 cases of ductal carcinoma *in situ* (DCIS), has been examined for mutation of *TP53*, allelic imbalance (AI) on 17p13, and expression of *TP53*, in a number of cases, has been studied using immunohistochemistry. Areas of DCIS, with or without adjacent invasive or benign cells, have been separately microdissected from paraffin-embedded sections and analysed by PCR for genetic changes to chromosome 17p13. *TP53* mutations and AI on 17p have been identified in cases of 'pure' DCIS as well as those with associated invasive carcinoma and, furthermore, have been identified in well-differentiated lesions as well as poorly differentiated ones.

**Keywords:** ductal carcinoma *in situ*; microdissection; *TP53*; immunohistochemistry; allelic imbalance

The identification of somatic mutations and chromosomal abnormalities occurring in human breast cancer has resulted in an increasingly complex picture of breast tumour development. The difficulty in distinguishing between genetic alterations, which represent key changes, and those which have been randomly acquired, has precluded the identification of events occurring at specific stages of breast tumorigenesis. This is largely owing to the failure of previous studies to focus on different histological subtypes, in particular early-stage breast tumours. However, in contrast to colorectal cancer, where there exists a series of well-defined preinvasive lesions, there is no clear precursor to invasive breast carcinoma. Furthermore, the classification of many early-stage breast lesions is often ambiguous.

A number of epidemiological studies on the risk of developing invasive carcinoma in patients with benign epithelial hyperplasia (Dupont and Page, 1985; Page *et al.*, 1985; Tavassoli and Norris, 1990) and with carcinoma *in situ* (Rosen *et al.*, 1980; Page *et al.*, 1982) do, however, suggest a possible continuum from proliferative epithelial changes of the breast to invasive carcinoma. Ductal carcinoma *in situ* (DCIS) is one of the earliest recognisable forms of breast cancer. Although cytologically the cells are often indistinguishable from those of invasive carcinoma, they are confined within the basement membrane surrounding the duct and there is no evidence of stromal invasion. As a result of its frequent finding adjacent to invasive carcinoma, and the observation that patients with DCIS have a risk ten times that of the general population of developing invasive breast tumours, often at the site of the original tumour, DCIS appears to be a likely candidate for a precursor lesion of invasive carcinoma (Betsill *et al.*, 1978; Page *et al.*, 1982, 1995). However, despite its high occurrence, the relationship between DCIS and invasive carcinoma remains unclear. This is largely due to the heterogeneous nature of the lesions which constitute DCIS. The disease consists of three main histological subtypes–comedo, cribriform and micropapillary–which differ in their biological behaviour and prognosis (Page and Rogers, 1987). In view of this a new classification has been proposed, dividing cases into well, intermediate and poor differentiation (Holland *et al.*, 1994). In addition, studies on the use of local excision as a possible treatment for DCIS suggest that those patients with the comedo subtype may be at greater risk of local recurrence (Lagios *et al.*, 1982). That these tumours may represent a more aggressive form of DCIS is also suggested by the findings that alterations to both c-*erb*B-2 (Gusterson *et al.*, 1988; Van de Vijver *et al.*, 1988; Barnes *et al.*, 1992*a)* and *TP53* (Varley *et al.*, 1991; Walker *et al.*, 1991; Poller *et al.*, 1993) have to date only been identified in the comedo subtype. However, most of these studies have been carried out on small numbers of tumours by immunohistochemical analysis, and in the case of the *TP53* gene, there are few reports in which DNA sequencing was used to confirm the presence of mutations in those cases of comedo DCIS showing positive staining (Davidoff *et al.*, 1991; O'Malley *et al.*, 1994). In addition allelic imbalance (AI), thought to represent inactivation of tumour-suppressor genes, has been demonstrated at a number of loci, including *TP53*, not only in comedo DCIS but also in cribriform and micropapillary variants of lower nuclear grade (Radford *et al.*, 1993; Munn *et al.*, 1995, 1996; Radford *et al.*, 1995; Stratton *et al.*, 1995; Zhuang *et al.*, 1995).

Here, we report the identification of *TP53* mutations in a panel of 35 cases of DCIS representing all three major histological subtypes, both with and without associated benign and invasive disease. In a number of cases we correlate the results with immunohistochemical staining and AI data, as the relationship between these and mutation of the *TP53* gene in breast tumours is unclear. Comparison of the patterns of alterations observed in different stages of tumour from the same patient allows us to draw important conclusions regarding the stepwise progression of breast tumours.

### Materials and methods

### *Tumour samples*

Nineteen cases of DCIS were obtained from Glenfield Hospital, Leicester, three of which had an associated area of frank invasive carcinoma. A further 17 cases were obtained from Christie Hospital, Manchester, consisting of 13 cases with DCIS and invasive carcinoma, three cases with an additional benign proliferative component, DCIS and invasive carcinoma, and one case with a benign component and invasive carcinoma. A total of 35 cases of DCIS were, therefore, available for study. For all cases the histological classification (given in Table I) was confirmed by a histopathologist (RAW and LM). Nuclear grade of DCIS was assessed according to Holland *et al.* (1994). All tumour samples were formalin fixed and paraffin embedded, and for the majority of cases a block containing normal breast tissue was also available.

Correspondence: JM Varley
Received 8 January 1996; revised 21 May 1996; accepted 28 May 1996

*Microdissection and DNA extraction*

Normal tissue, areas of DCIS from within single ducts, and benign or invasive carcinoma where present, were microdissected from single dewaxed, haematoxylin-stained 5 or 10 μm sections, and DNA was extracted as previously described (Munn *et al.*, 1995). In all cases epithelial cells were microdissected and analysed, and there was minimal contamination by stromal or inflammatory cells. An average of three consecutive sections per tumour were analysed.

*PCR amplification*

PCR reactions were carried out in a volume of 10 μl, containing 200 μM each dNTP (Promega), 10 pmol of each primer, 0.5 units *Taq* DNA polymerase (Promega) and 1 μl DNA extracted from the microdissected tissue. All reactions were amplified for 35 cycles, including an initial denaturation step at 94°C for 4 min, and a final extension at 72°C for 10 min. For the markers described, each cycle consisted of denaturation at 94°C and extension at 72°C for 1 min each. Annealing was carried out for 1 min at temperatures ranging from 56–66°C. However, a number of markers were amplified using a Touchdown PCR (Don *et al.*, 1991), in which two cycles were performed at an initial annealing temperature of 70°C. The annealing temperature was then reduced by increments of 2°C, and two cycles were carried out at each temperature until 50°C was reached, at which 20 cycles were carried out.

**Table I** Histology of tumours studied

| *Case no.* | *Histology* | *Nuclear grade*[a] |
|---|---|---|
| Series 1. DCIS | | |
| 6 | Comedo | High |
| 15 | Cribriform/micropapillary | Low |
| 34 | Comedo | High |
| 56 | Comedo/micropapillary | Intermediate |
| 75 | Comedo | High |
| 106 | Cribriform | Low |
| 144 | Comedo/cribriform | Intermediate |
| 257 | Comedo/cribriform | High |
| 1886 | Cribriform | Low |
| 2281 | Comedo | High |
| 3410 | Comedo | High |
| 3800 | Comedo/cribriform | High |
| 3805 | Comedo | High |
| 4119 | Comedo | High |
| 4736 | Comedo | Intermediate |
| 4753 | Comedo | High |
| Series 2. DCIS and invasive carcinoma | | |
| 452 | Micropapillary + ID | Low (II) |
| 458 | Cribriform + ID | High (III) |
| 593 | Cribriform + ID | Low (I) |
| 982 | Cribriform + ID | Intermediate (II) |
| 1141 | Cribriform + ID | ND (III) |
| 1565 | Florid hyperplasia, comedo + ID | Intermediate (II) |
| 2753 | Cribriform/solid + ID | Low (II) |
| 2822 | Cribriform + ID | Low (II) |
| 2931 | Micropapillary + ID | ND (III) |
| 2939 | Cribriform + ID | Intermediate (II) |
| 2996 | Sclerosing adenosis, comedo + ID | High (III) |
| 3041 | Cribriform + tubular carcinoma | Low (I) |
| 4410 | Comedo + ID | High (III) |
| 4617 | Comedo/micropapillary + ID | High (III) |
| 4681 | Cribriform/papillary + ID | Intermediate (II) |
| 5170 | Comedo + ID | High (III) |
| 6045[b] | Papilloma, comedo + ID | Intermediate (II) |
| 6256 | Cribriform + ID | Low (II) |
| 6384 | Comedo + ID | Intermediate (II) |
| 6457 | Fibroadenosis, comedo + ID | High (III) |

Series 1 comprises those samples in which DCIS is present alone, series 2 in which there is an additional benign or invasive component. [a]Nuclear grade of DCIS was classified according to Holland *et al.* (1994) as low, intermediate or high. For samples in series 2, the grade of the invasive component is given in parentheses where known, and was assessed according to Elston and Ellis (1991). [b]Sample 6045 was classified as comedo DCIS adjacent to invasive, with papilloma present. On the section we obtained, there was no DCIS, therefore only the papilloma and invasive cell were studied. ND, not determined.

*Single-strand conformation polymorphism analysis*

Tumour DNA was screened for mutations in exons 4–9 of the *TP53* gene using single-strand conformation polymorphism (SSCP) (Orita *et al.*, 1989). For exon 4 and 9 the following primers were used:

| | |
|---|---|
| Exon 4 | 5′-TCTGGTAAGGACAAGGGTT-3′ |
| | 5′-GGCAACTGACCGTGCAAG-3′ |
| Exon 9 | 5′-ACTAAGCGAGGTAAGCAA-3′ |
| | 5′-CTTTCCACTTGATAAGAGG-3′ |

For exons 5–8 two sets of primers were used, as it is thought that the position of a mutation within a fragment may influence its detection (Sheffield *et al.*, 1993). The sequences of these primers have previously been described (Varley *et al.*, 1991). The majority of the primers used amplified products of between 100 and 250 base pairs, as this size is thought to be optimal for the detection of mutations by SSCP. For larger sequences products were digested by restriction endonucleases before gel electrophoresis. Products were radiolabelled by reducing the concentration of cold dCTP in the reaction to 10 μM and adding 1 μCi [α-$^{32}$P]dCTP. Before electrophoresis, 2 μl product were diluted with 6 μl 10 mM EDTA/0.1% sodium dodecyl sulphate (SDS) and 6 μl loading dye (95% formamide, 20 mM EDTA, 0.05% bromophenol blue, 0.05% xylene cyanol). Samples were heat denatured for 2 min and immediately loaded on a non-denaturing polyacrylamide gel run in 0.5 × Tris-borate buffer. Products were analysed normally on several types of gel: 0.5 × MDE HydroLink (AT Biochemicals) or 8% polyacrylamide, containing 5 or 10% glycerol. Gels were either run at 15W for 8–10 h at room temperature with the use of a cooling fan, or at 4°C. After electrophoresis, gels were transferred to 3MM paper, dried at 80°C under vacuum and exposed to radiographic film.

*DNA sequencing*

Normal and tumour DNA were amplified for 35 cycles using the outer most set of primers. A secondary reaction in a volume of 30 μl, containing 0.5 μl of first round product and 5 pmol of each primer, was then amplified for twenty cycles. Products were analysed on 2% agarose gels and the fragment subsequently purified using Magic DNA Clean-up Columns (Promega). Double-stranded PCR products were sequenced using a variation of the dideoxy chain termination method (Winship, 1989). Products were analysed on 6% denaturing polyacrylamide gels.

*Immunohistochemistry*

Paraffin-embedded tissue sections of 5 μM thickness were stained using both DO-1 (a gift from D Lane), a murine monoclonal antibody which recognises a denaturation-resistant epitope at amino acids 20–25 of human p53, and CM-1 (Novocastra Labs), a rabbit polyclonal serum raised against recombinant human p53 protein (Midgley *et al.*, 1992; Vojtesek *et al.*, 1992). Sections were incubated with primary antibody (undiluted and 1:800 dilutions respectively) overnight at 4°C. For both antibodies the avidin–biotin–peroxidase complex was developed using 3,3-diaminobenzidine as the chromogen. Only nuclear staining was assessed. Tumours were subdivided into those with >50% cells reactive; 20–50% positive cells; patchy staining in some ducts <20%; scant positive cells and negative. Only samples from series 1 were analysed by immunohistochemistry.

*Detection of allelic imbalance*

Four microsatellite DNA length polymorphisms on chromosome 17p were used to detect AI: D17S926 (Gyapay *et al*., 1994), *TP53*(AAAAT)$_n$ (Futreal *et al*., 1992), *TP53*(CA)$_n$ (Jones and Nakamura, 1992) and D17S513 (Oliphant *et al*., 1991). The primer sequences used to amplify the pentanucleotide and dinucleotide repeats within *TP53* differed from those used in the original publications.

| | |
|---|---|
| *TP53*(AAAAT)$_n$ | 5'-AAACAGCTCCTTTAATGGCAG-3' |
| | 5'-ATCATTTGAATCCGGGAGGA-3' |
| *TP53*(CA)$_n$ | 5'-CCTGAGGATACTATTCAGCC-3' |
| | 5'-CCCACAGAGCGAGACTGTCT-3' |

In addition, four polymorphisms were used to control for AI on the long arm of chromosome 17, as previously described (Munn *et al*., 1996). Products were radiolabelled by incorporation of [α-$^{32}$P]dCTP, and analysed on 6% denaturing gels. For analysis of the *Bst*U1 restriction fragment length polymorphism (RFLP) in exon 4 (Ara *et al*., 1991), normal and tumour DNA were amplified in a 30 μl volume, and 10 μl of product was subsequently digested. Products were analysed on 3% agarose gels. AI was determined by eye in heterozygous patients by comparing the ratio of the two alleles in the tumour and normal samples. A difference in intensity between the two alleles in the tumour, which by eye appeared to be at least 2-fold, was taken to represent AI. For microsatellite repeat polymorphisms a number of cases were also confirmed by the use of a phosphorimager (Molecular Dynamics).

### Results

*Mutations in the* TP53 *gene*

Exons 4–9 of the *TP53* gene were amplified individually from normal tissue and several areas of tumour from all 36 cases, and subsequently analysed by SSCP. This region encodes the most highly conserved domains of the protein, and is the site of the majority of mutations that have been identified in human tumours. Seven possible somatic mutations were identified in exons 5–8 by the presence of additional bands, or bands of aberrant mobility in the tumour sample (Figure 1a). For all positive samples, the SSCP analysis was repeated from reamplified material to ensure that the altered pattern of bands was not caused by extraneous DNA, or by the misincorporation of a base in the initial cycles of the PCR amplification. In all cases the results were reproducible. In five cases the mutations were confirmed by DNA sequencing of double-stranded PCR products (Table II, Figure 1b). Four of the mutations were base substitutions, while the remaining mutation was a frameshift due to a single base deletion. Three of these cases (6, 2281 and 3805) were comedo DCIS of poor differentiation. However, in one case, 106, the mutation was present in a well-differentiated lesion. For cases 106, 2281 and 3805 more

**Figure 1** (**a**), Detection of *TP53* mutation by SSCP analysis of $^{32}$P-labelled PCR products. Bands of aberrant mobility in samples 106 and 2281, suggestive of a mutation, are indicated by arrows. The examples shown were run at room temperature on a 5% polyacrylamide gel containing 10% glycerol, and on a 0.5 × MDE polyacrylamide gel containing 5% glycerol (exons 5 and 7 respectively). In this autoradiographic exposure no signal can be seen from sample 1886Cr. (**b**), Mutations detected by SSCP analysis were confirmed by sequencing of double-stranded PCR products; sequence changes are indicated. In case 2281 the mutation is shown on the complementary strand. In case 458 no mutation was detected in the DCIS component; however, the mutation in the invasive sample appears to be homo- or hemizygous. In contrast, the presence of the wild-type sequence, of roughly equal intensity, in case 2281 suggests that the mutation is heterozygous. N, normal; C, comedo DCIS; Cr, cribriform DCIS; I, invasive tumour.

**Table II** *TP53* mutations identified in this study and the corresponding results of immunohistochemical staining and AI studies

| *Case* | *Histology* | *Grade* | *Exon* | *Codon* | *Base change* | *Amino acid change* | *IHC*[a] | *AI*[b] |
|---|---|---|---|---|---|---|---|---|
| 106 | Cribriform | Low | 5 | 153 | CCC→CCA | Pro→Pro | +/– | + |
| 6 | Comedo | High | 6 | 202 | CGT→CAT | Arg→His | – | + |
| 458 | Invasive | III | 6 | 215 | AGT→GGT | Ser→Gly | ND | – |
| 2281 | Comedo | High | 7 | 237 | ATG→ATC | Met→Ile | + + | – |
| 3805 | Comedo | High | 8 | 304 | Del T | Terminates at 344 | + + | + |

[a]IHC, immunohistochemistry; + +, strong nuclear staining in at least 50% of the tumour cells; +/–, heterogeneous staining; ND, not done. [b]AI, allelic imbalance determined at the *TP53* locus using three intragenic polymorphisms.

**Table III** p53 immunohistochemical staining data for 15 cases of DCIS without associated invasive disease

| *Case* | *Histology* | *Grade* | *p53 staining*[a] |
|---|---|---|---|
| 6 | Comedo | High | – |
| 15 | Cribriform/micropapillary | Low | – |
| 56 | Comedo/micropapillary | Intermediate | –/+ |
| 75 | Comedo | High | + + |
| 106 | Cribriform | Low | +/–[b] |
| 144 | Comedo/cribriform | High | + |
| 257 | Comedo/cribriform | High | + + |
| 1886 | Cribriform | High | – |
| 2281 | Comedo | High | + + |
| 3410 | Comedo | High | – |
| 3800 | Comedo/cribriform | High | – |
| 3805 | Comedo | High | + + |
| 4119 | Comedo | High | – |
| 4736 | Comedo/micropapillary | Intermediate | – |
| 4753 | Comedo | High | –/+ |

[a]+ +, nuclear staining in more than 50% of the tumour cells; +, nuclear staining in 20–50% of the tumour cells; +/–, patchy pattern of positive staining; –/+, scant positive staining. [b]Faint cytoplasmic staining with CM-1.

than one DCIS-containing duct from the same quadrant was analysed and the mutation was found to be present in some ducts but not others, indicating that the tumour is heterogeneous. The remaining case, 458, showed no evidence of a mutation in the two areas of cribriform DCIS studied, but did contain a mutation in the infiltrating carcinoma. In cases 6, 458 and 3805, the DNA sequence of the tumour showed only mutant sequence, indicating that loss of the remaining wild-type allele had occurred. Two cases, one consisting of DCIS and the other of cribriform DCIS with infiltrating carcinoma, gave positive results in the SSCP analysis of exon 7 which could not be confirmed by DNA sequencing (cases 3800 and 2753). In addition, eight cases, which appeared negative by SSCP (of which four were invasive tumours), including one which showed positive staining with p53 antibodies (see below), were also sequenced and found not to contain any mutations in the region analysed.

### *p53 expression*

Fifteen cases of pure DCIS from series 1 were stained with the antibodies DO-1 and CM-1 (see Table III). In all areas of normal tissue negative staining patterns were observed. Of those eight cases (53%), which showed positive staining, the patterns of staining could be divided into four classes. The majority of those that were positive showed moderate to strong nuclear staining, which was present in at least 50% of the tumour cells (Figure 2). This pattern was observed predominantly in comedo DCIS, but was also seen in a poorly differentiated area of cribriform DCIS in case 257. In a further case showing positive staining, the proportion of positive cells was as low as 30%. In addition, three cases displayed a heterogeneous pattern of staining in which tumour cells in some parts of the tumour showed positive nuclear staining, while others were negative. One cribriform

**Figure 2** Immunohistochemical staining for *TP53* expression using polyclonal antiserum CM-1. (**a**), Case 106 shows strong nuclear staining in scattered cells in an area of cribriform DCIS. Case 2281 (**b**), in which a mutation in exon 7 of the *TP53* gene was identified, shows strong nuclear staining in an area of comedo DCIS.

case also showed some cytoplasmic staining with CM-1; however, cross-reactivity of this antibody has been reported previously (Cornelis *et al.*, 1994). Of the cases stained, four contained a *TP53* mutation. One case (6), which contained a missense mutation, had no evidence of staining. Case 2281, which had a missense mutation, and case 3805, which had a frameshift resulting in deletion of the last 50 amino acids, both showed strong nuclear staining (Figure 2b). However, case 106, which had silent mutation, also showed positive staining, although the pattern was patchy (Figure 2a). In addition, for five cases in which no mutation was identified by SSCP, one of which was also sequenced, positive staining was observed.

Series 1

| Case | **6** | 15 | 34 | 56 | 75 | **106** | 144 | 257 | 1886 | **2281** | 3410 | 3800 | **3805** | 4119 | 4736 | 4753 |
|---|---|---|---|---|---|---|---|---|---|---|---|---|---|---|---|---|
| Locus | **C1** C2 | MP | C | MP | C1 C2 | **Cr1** Cr2 | C1 C2 | C | Cr | **C** | C | Cr | **C** | C | C1 C2 | C1 C2 |
| D17S926 | ● | | – | ○ | ○ | ● | | | – | ○ | ● | | ○ | | | |
| D17S513 | | | | | | | ○ ○ | ○ | ○ | | ○ | ○ | ○ | ○ | ● | ● |
| *TP53*† | ● ○ | ○ | ○ | ● | ● ● | ● ● | ● ● | ● | ○ | ○ | – | – | ● | ● | ● ○ | ● ○ |

Series 2

| Case | 452 | **458** | 593 | 982 | 1141 | 1565 | 2753 | 2822 | 2931 | 2939 | 2996 | 3041 | 4410 | 4617 | 4681 | 5170 | 6045 | 6256 | 6384 | 6457 |
|---|---|---|---|---|---|---|---|---|---|---|---|---|---|---|---|---|---|---|---|---|
| Locus | MP I | Cr I | Cr I | Cr I | Cr I | B Cl C 2 I | Cr I | Cr I | MP I | Cr I | B C I | Cr I | C I | MP I | P Cr I | C I | B I | Cr I | C I | B C I |
| D17S926 | – | | – | ● ● | | | | ● ● | ○ ○ | | | | | | ● | ○ ○ | | | | |
| D17S513 | ● ● | | | | ○ ○ | ○ ○ ○ ○ | | – | | | ○ ● ● | ● ● | ○ ○ | ○ ● | – | | ○ ○ | – | ○ ○ | ○ ○ |
| *TP53*† | ● ● | ○ ○ | ○ ○ | ● ● | ● ● | ○ ● ○ ● | ○ ○ | – | – | ○ ○ | ○ ● ● | ● ● | – | – | ○ ○ ● | ● ● | – | ● ● | – | ● ● ● |

**Figure 3** Results of allelic imbalance (AI) studies at chromosome 17p13. (○), informative and both alleles retained; (●), informative and showing AI; (–), uninformative; no symbol, not tested owing to lack of material or sample consistently failing to amplify. † AI at *TP53* was determined using three intragenic polymorphisms in order to maximise the number of informative cases. Cases in bold contain a *TP53* mutation. C, comedo DCIS; Cr, cribriform DCIS; MP, micropapillary DCIS; P, papillary DCIS; B, benign; I, invasive. Where multiple ducts have been sampled from the same patient they are indicated by Crl, Cr2, etc.

### *AI at chromosome 17p13*

AI at *TP53* was determined by the use of three intragenic polymorphisms. In order to distinguish between events occurring at the *TP53* locus from those involving the telomeric region of 17p, two distal markers, D17S513 and D17S926, were used. Several markers on 17q were also used, as previously described (Munn *et al.*, 1996), and serve as a control for possible loss of a whole copy of chromosome 17. As a result of limiting amounts of DNA from some lesions, it was not possible to test all cases with every marker. In addition, some cases consistently failed to amplify with some sets of primers. All cases were, however, informative for at least one marker on 17p and, in 21 of the 35 DCIS cases (60%), AI on 17p was observed in the DCIS component (Figure 3). These cases included five well-differentiated, cribriform or micropapillary lesions. Eight of these cases showed AI at all informative markers studied on both 17p and 17q, suggesting possible loss of a copy of the chromosome, while a further two cases showed possible loss of a copy of the short arm. Of 28 cases of DCIS that were informative at the *TP53* locus, 19 cases (68%) of DCIS showed AI. However, only ten of these cases showed retention of either of the distal markers used. As there is evidence for a second tumour-suppressor gene, which maps somewhere between these two markers, then the percentage of DCIS cases showing AI, which appears to target *TP53*, is 36%. A single case of comedo DCIS, 3410, was found to show AI independent from *TP53* at D17S926, implicating the more distal gene in early breast tumours. Of those cases known to contain *TP53* point mutations, three were predicted to show AI based on the sequencing data. Both cases 6 and 3805 showed clear loss of an allele. However, case 458 showed retention of heterozygosity at *TP53*. This discordancy in the results can be explained if a deletion of the wild-type homologue or intragenic recombination had occurred between the $(AAAAT)_n$ repeat in intron 1 and the site of the mutation in exon 6. Both cases 2281 and 106 had sequences suggesting that the mutation was heterozygous. The AI data confirmed this for case 2281, but case 106 showed AI suggesting that the residual wild-type signal in the sequence was due to normal contamination. Of those cases studied with an associated area of invasive carcinoma, 12 showed AI in either component and, in ten of these cases, there was the same pattern of allele loss in both. The remaining two cases showed AI in the invasive tumour but not in the DCIS (4617 and 4681, Figure 4a). Both these samples showed loss at other loci in the DCIS components. Case 4617 showed loss of 17q markers (Munn *et al.*, 1996) and the cribriform component of 4681 showed loss of chromosome 1 markers (Munn *et al.*, 1995). However, the papillary component of 4681 showed no loss at any of the loci we have studied. For four cases, associated benign proliferative disease was also studied. In two of these cases, no AI was observed, even though there was AI in the DCIS and invasive carcinoma from the same patient. However, in case 6457 AI was observed in an area of fibroadenosis. Loss of the same allele was demonstrated in both the DCIS and invasive components. There was an area of atypical ductal hyperplasia described in the original report on 6457, but we did not receive this material and, therefore, could not analyse it. In eight cases DCIS from within separate ducts on the same section were analysed. Consistent with the results from the mutation analysis, four of these showed a different pattern of AI in each component (Figure 4b), which in one case, 106, involved the loss of different alleles.

**Figure 4** Allelic imbalance (AI) at the $(AAAT)_n$ microsatellite repeat polymorphism in the *TP53* gene. (**a**), AI in the invasive component of case 4681, but not in the DCIS. (**b**), AI in an area of comedo DCIS from patient 1565. In contrast, a second area of comedo DCIS present on the same section showed retention of both alleles. N, normal; C, comedo DCIS; Cr, cribriform DCIS; I, invasive tumour.

### Discussion

To date, there have been few reports documenting the identification of genetic alterations in DCIS, as the small size of the lesions restricts analysis to paraffin-embedded material. However, the use of PCR to analyse small microsatellite repeat polymorphisms has resulted in a number of reports of AI at several regions of the genome, thought to harbour tumour-suppressor genes. Here, we have used a combination of tissue microdissection and PCR to look for *TP53* mutations and AI on chromosome 17p13. In contrast to many of the previously published studies, we have used microdissection to isolate small numbers of tumour cells from within single DCIS-containing ducts rather than from larger areas of DCIS, which may consist of several ducts. This overcomes problems of stromal contamination and tumour heterogeneity, which may mask AI.

The identification of *TP53* mutations and AI at 17p13 in cases of DCIS, both with and without associated invasive carcinoma, suggests that these are important changes in the early stages of breast tumorigenesis. Previous estimates of the frequency of *TP53* mutations in invasive carcinomas, based on immunohistochemistry, have ranged from 25–60% (for review see Eeles *et al.*, 1994). Molecular analysis has, in contrast, revealed a frequency of only 13–30% (Prosser *et al.*, 1990; Varley *et al.*, 1991; Cornelis *et al.*, 1994). In DCIS the reported frequency of p53 overexpression is much lower at around 10–25% (Bartek *et al.*, 1990; Poller *et al.*, 1993), although Bobrow *et al.* (1995) reported 50%, and there are only two reported cases in which *TP53* mutations were identified in DCIS by sequencing (Davidoff *et al.*, 1991; O'Malley *et al.*, 1994). Using a combination of SSCP and DNA sequence analysis of small foci of tumour cells from DCIS, we have identified *TP53* mutations in four cases (11%). Those three cases in which the mutation was missense or led to a truncated protein, were all comedo DCIS of poor differentiation. The fourth case, a well-differentiated cribriform lesion, contained a silent mutation. This case has previously been reported to show AI on chromosome 1 (Munn *et al.*, 1995). Therefore, selection for a mutation in the gene, which is the target of AI on chromosome 1, may account for the maintenance of this *TP53* mutation in the tumour. In 53% of those cases which were stained with anti-p53 antibodies, positive staining was observed. This frequency is significantly higher than seen in previous studies, possibly because of our small sample size. However, the majority of these cases did not appear to have *TP53* mutations. A likely explanation for this may be the failure of SSCP to identify all mutations, although in previous studies of known *TP53* mutants, its sensitivity is estimated to be around 90% (Moyret *et al.*, 1994). Also, the possibility of mutations in the regulatory region of the gene or in the remainder of the coding region cannot be eliminated. In addition, some of these cases showed staining in a small proportion of the cells in the tumour, or showed a heterogeneous pattern of staining. In such cases the presence of a mutation in a wild-type background may be difficult to detect using PCR-based techniques. Furthermore, it has been reported that overexpression of TP53 protein does occur in the absence of mutation (Barnes *et al.*, 1992*b*). This may be a result of the overexpression of a protein which binds p53 and stabilises it, e.g. MDM2, or it may be caused by the presence of DNA damage in the tumour cell. Whatever the mechanism of the observed overexpression, these results are interesting in that there are no previous reports of positive staining in cribriform DCIS, and there is only one reported case of micropapillary DCIS that shows overexpression of *TP53* (Thor *et al.*, 1992).

In contrast, the observed frequency of AI on chromosome 17p in DCIS is similar to that reported for invasive tumours (Varley *et al.*, 1991; Andersen *et al.*, 1992; Cornelis *et al.*, 1994). In a number of cases the *TP53* gene appears to be the target of AI. However, few of these cases were found to contain a *TP53* mutation. The loss of one copy of the *TP53* gene may, therefore, confer some growth advantage on the cell, possibly because the reduced dosage of wild-type p53 protein increases the tumour's chances of accumulating further mutations. In a larger number of cases there was also AI distal to *TP53*. There is both molecular and functional evidence for a tumour-suppressor gene distinct from *TP53* near the telomere of chromosome 17p (Stack *et al.*, 1995; Theile *et al.*, 1995). Therefore, it is unclear in these cases which gene was the target of AI; however, one case did show AI at 17p13.3 independent from *TP53*, suggesting that this gene may have a role in the early stages of breast tumorigenesis. A number of these cases also showed AI at all informative markers studied on 17q, and in the majority the patterns consisted of clear allele loss rather than allele imbalance, suggesting possibly that a copy of the whole chromosome had been lost. Obviously, using PCR-based techniques it cannot easily be determined whether this has been followed by duplication of the remaining homologue, but previous studies, using interphase cytogenetics, have reported polysomy for chromosome 17 in DCIS (Murphy *et al.*, 1995).

From these data, and much of that which has previously been documented, it is evident that a large number of the genetic alterations known to occur in invasive breast tumours are already present in DCIS. This is clearly not only the case for high-grade comedo lesions. Here we have demonstrated AI on chromosome 17p in a number of well-differentiated cribriform and micropapillary lesions, both with and without associated invasive disease. Unfortunately, we failed to identify common *TP53* mutations in DCIS and invasive tumour from the same patient, which would have provided strong evidence for the preinvasive nature of DCIS. The patterns of AI in a number of cases do, however, support this. We have also identified one case of fibroadenosis, which shows AI and displays the same pattern of allele loss in DCIS and invasive components from the same patient (6457). The relationship between benign, *in situ* and invasive disease is not always likely to be as simple as in 6457. Although DCIS has previously been reported to be clonal in origin (Noguchi *et al.*, 1994), there is clearly heterogeneity within the DCIS component, and we and others have previously reported cases in which there was loss in the benign or *in situ* component and not in the invasive tumour (Munn *et al.*, 1995, 1996; Stratton *et al.*, 1995). It is likely, therefore, that DCIS exists as divergent populations, only some of which may acquire the changes necessary for them to become invasive carcinoma. The application of this kind of approach to lesions, such as atypical ductal hyperplasia, which are thought to be borderline between hyperplasia and neoplasia, will hopefully provide a clearer picture of the pathway of tumour development.

**Acknowledgements**
We are grateful to Sheila Dearing for technical help and to Nigel Barron for assistance with photographic work. This work was financially supported by the Cancer Research Campaign, KEM was in receipt of a Medical Research Council studentship.

### References

ANDERSEN TI, GAUSTAD A, OTTESTAD L, FARRANTS GW, NESLAND JM, TVEIT KM AND BORRESEN A-L. (1992). Genetic alterations of the tumour suppressor gene regions 3p, 11p, 13q, 17p and 17q in human breast carcinomas. *Genes, Chrom. Cancer*, **4,** 113–121.

ARA S, LEE PSY, HANSEN MF AND SAYA H. (1991). Codon 72 polymorphism of the TP53 gene. *Nucleic Acids Res.*, **18,** 4961.

BARNES DM, BARTKOVA J, CAMPLEJOHN RS, GULLICK WJ, SMITH PJ AND MILLIS RR. (1992*a*). Overexpression of the c-*erb*B-2 oncoprotein: why does this occur more frequently in ductal carcinoma *in situ* than in invasive mammary carcinoma and is this of prognostic significance? *Eur. J. Cancer*, **28,** 644–648.

BARNES DM, HANBY AM, GILLET CE, MOHAMMED S, HODGSON S, BOBROW LG, LEIGH IM, PURKIS T, MACGEOCH C, SPURR NK, BARTEK J, VOJTESEK B, PICKSLEY SM AND LANE DP. (1992*b*). Abnormal expression of wild-type p53 protein in normal cells of a cancer family patient. *Lancet*, **340,** 259–263.

BARTEK J, BARTKOVA B, VOJTESEK B, STASKOVA Z, REJTHAR A, KOVARIK J AND LANE DP. (1990). Patterns of expression of the p53 tumour suppressor in human breast tissues and tumours *in situ* and *in vitro*. *Int. J. Cancer*, **46,** 839–844.

BETSILL WL, ROSEN PP, LIEBERMAN PH AND ROBBINS GF. (1978). Intraductal carcinoma: long-term follow-up after treatment by biopsy alone. *J. Am. Med. Ass.*, **239,** 1863–1867.

BOBROW LG, HAPPERFIELD LC, GREGORY WM AND MILLIS RR. (1995). Ductal carcinoma *in situ*: assessment of necrosis and nuclear morphology and their association with biological markers. *J. Pathol.*, **176,** 333–341.

CORNELIS RS, VAN VLIET M, VOS CB, CLETON-JANSE A-M, VAN DER VIJVER MJ, PETERSE JL, KHAN PM, BORRESEN A-L, CORNELISSE CJ AND DEVILEE P. (1994). Evidence for a gene on 17p13.3, distal to TP53, as a target for allele loss in breast tumours without p53 mutations. *Cancer Res.*, **54,** 4200–4206.

DAVIDOFF AM, KERNS B-JM, IGLEHART JD AND MARKS JR. (1991). Maintenance of p53 alterations throughout breast cancer progression. *Cancer Res.*, **51,** 2605–2610.

DON RH, COX PT, WAINWRIGHT BJ, BAKER K AND MATTICK JS. (1991). 'Touchdown' PCR to circumvent spurious priming during gene amplification. *Nucleic Acids Res.*, **19,** 4008.

DUPONT WD AND PAGE DL. (1985). Risk factors for breast cancer in women with proliferative breast disease. *N. Engl. J. Med.*, **312,** 146–151.

EELES RA, BARTKOVA J, LANE DP AND BARTEK J. (1994). The role of p53 in breast cancer development. *Cancer Surveys*, **18,** 57–75.

ELSTON CW AND ELLIS IO. (1991). Pathological prognostic factors in breast cancer. I. The value of histological grade in breast cancer: experience from a large study with long-term follow-up. *Histopathology*, **19,** 403–410.

FUTREAL AP, BARRETT JC AND WISEMAN RW. (1992). An Alu polymorphism intragenic to the TP53 gene. *Nucleic Acids Res.*, **19,** 6977.

GUSTERSON BA, MACHIN LG, GULLICK WJ, GIBBS NM, POWLES TJ, ELLIOTT C, ASHLEY S, MONAGHAN P AND HARRISON S. (1988). c-*erb*B-2 expression in benign and malignant breast disease. *Br. J. Cancer*, **58,** 453–457.

GYAPAY G, MORISETTE J, VIGNAL A, DIB C, FIZAMES C, MILLASSEAU C, MARC S, BERNARDI G, LATHROP M AND WEISSENBACH J. (1994). The 1993–1994 Genethon human genetic linkage map. *Nature Genet.*, **7,** 246–339.

HOLLAND R, PETERSE JL, MILLIS RR, EUSEBI V, FAVERLY D, VAN DER VIJVER MJ AND ZAFRANI B. (1994). Ductal carcinoma *in situ*: a proposal for a new classification. *Semin. Diag. Pathol.*, **11,** 167–180.

JONES MH AND NAKAMURA Y. (1992). Detection of loss of heterozygosity at the human TP53 locus using a dinucleotide repeat polymorphism. *Genes, Chrom. Cancer*, **5,** 89–90.

LAGIOS MD, WESTDAHL PR, MARGOLIN FR AND ROSE MR. (1983). Duct carcinoma *in situ*: relationship of extent of noninvasive disease to the frequency of occult invasion, multicentricity, lymph node metastases and short-term treatment failures. *Caner*, **50,** 1309–1314.

MIDGLEY CA, FISHER CJ, BARTEK J, VOJTESEK B, LANE DP AND BARNES DM. (1992). Analysis of p53 expression in human tumours: an antibody raised against human p53 expressed in *Escherichia coli*. *J. Cell Sci.*, **101,** 183–189.

MOYRET C, THEILLET C, LAURENT P, MOLES J-P, THOMAS G AND HAMELIN R. (1994). Relative efficiency of denaturing gradient gel electrophoresis and single strand conformation polymorphism in the detection of mutations in exons 5 to 8 of the p53 gene. *Oncogene*, **9,** 1739–1743.

MUNN KE, WALKER RA AND VARLEY JM. (1995). Frequent alterations of chromosome 1 in ductal carcinoma *in situ* of the breast. *Oncogene*, **10,** 1653–1657.

MUNN KE, WALKER RA, MENASCE L AND VARLEY JM. (1996). Allelic imbalance in the region of the *BRCA1* gene in ductal carcinoma *in situ* of the breast. *Br. J. Cancer*, **73,** 636–640.

MURPHY DS, MCHARDY P, COUTTS J, MALLON EA, GEORGE WD, KAYE SB, BROWN R AND KEITH WN. (1995). Interphase cytogenetic analysis of *ERB*B-2 and *TOPO*IIa co-amplification in invasive breast cancer and polysomy of chromosome 17 in ductal carcinoma *in situ*. *Int. J. Cancer*, **64,** 18–26.

NOGUCHI S, MOTOMURA K, INAJI H, IMAOKA S AND KOYAMA H. (1994). Clonal analysis of predominantly intraductal carcinoma and precancerous lesions of the breast by means of polymerase chain reaction. *Cancer Res.*, **54,** 1849–1853.

OLIPHANT AR, WRIGHT EC, SWENSEN J, GRUIS NA, GOLDGAR D AND SKOLNICK MH. (1991). Dinucleotide repeat polymorphism at the D17S513 locus. *Nucleic Acid Res.*, **19,** 4794.

O'MALLEY FP, VNENCAK-JONES CL, DUPONT WD, PARL F, MANNING S AND PAGE DL. (1994). p53 mutations are confined to the comedo type ductal carcinoma *in situ* of the breast. Immunohistochemical and staining data. *Lab. Invest.*, **71,** 67–72.

ORITA M, SUZUKI Y, SEKIYA T AND HAYASHI K. (1989). Rapid and sensitive detection of point mutations and DNA polymorphisms using the polymerase chain reaction. *Genomics*, **5,** 874–879.

PAGE DL AND ROGERS LW. (1987). Carcinoma *in situ* (CIS). In *Diagnostic Histopathology of the Breast*, Page DL and Anderson TJ. (eds) pp. 157–192. Churchill-Livingstone: Edinburgh, UK.

PAGE DL, DUPONT WD, ROGERS LW AND LANDENBERGER M. (1982). Intraductal carcinoma of the breast: follow-up after biopsy only. *Cancer*, **49,** 751–758.

PAGE DL, DUPONT WD, ROGERS LW AND RADOS MS. (1985). Atypical hyperplastic lesions of the female breast: a long term follow-up study. *Cancer*, **55,** 2698–2708.

PAGE DL, DUPONT WD, ROGERS LW, JENSEN RA AND SCHUYLER PA. (1995). Continued local recurrence of carcinoma 15-25 years after a diagnosis of low grade ductal carcinoma *in situ* of the breast treated only by biopsy. *Cancer*, **76,** 1197–1200.

POLLER DN, ROBERTS EC, BELL JA, ELSTON CW, BLAMEY RW AND ELLIS IO. (1993). p53 protein expression in mammary ductal carcinoma *in situ*: relationship to immunohistochemical expression of estrogen receptor and c-*erb*B-2 protein. *Hum. Pathol.*, **24,** 463–468.

PROSSER J, THOMPSON AM, CRANSTON G AND EVANS HJ. (1990). Evidence that p53 behaves as a tumour suppressor gene in sporadic breast tumours. *Oncogene*, **5,** 1573–1579.

RADFORD DM, FAIR K, THOMPSON AM, RITTER JH, HOLT M, STEINBRUECK T, WALLACE M, WELLS SA AND DONIS-KELLER HR. (1993). Allelic loss on chromosome 17 in ductal carcinoma *in situ* of the breast. *Cancer Res.*, **53,** 2947–2950.

RADFORD DM, FAIR KL, PHILLIPS NJ, RITTER JH, STEINBRUEK T AND HOLT MS. (1995). Allelotyping of ductal carcinoma *in situ* of the breast: deletion of loci on 8p, 13q, 16q, 17p and 17q. *Cancer Res.*, **55,** 3399–3405.

ROSEN PP, BRAUN DW AND KINNE DE. (1980). The clinical significance of preinvasive breast carcinoma. *Cancer*, **46,** 919–925.

SHEFFIELD VC, BECK JS, KWITEK AE, SANDSTROM DW AND STONE EM. (1993). The sensitivity of single-strand conformation polymorphism for the detection of single base substitutions. *Genomics*, **16,** 325–332.

STACK M, JONES D, WHITE G, LISCIA DS, VENESIO T, CASEY G, CRICHTON D, VARLEY J, MITCHELL E, HEIGHWAY J AND SANTIBANEZ-KOREF M. (1995). Detailed mapping and loss of heterozygosity analysis suggest a suppressor locus involved in sporadic breast cancer within a distal region of chromosome band 17p13.3. *Hum. Mol. Genet.*, **4,** 2047–2055.

STRATTON MR, COLLINS N, LAKHANI SR AND SLOANE JP. (1995). Loss of heterozygosity in ductal carcinoma *in situ* of the breast. *J. Pathol.*, **175,** 195–201.

TAVASSOLI FA AND NORRIS HJ. (1990). A comparison of the results of long-term follow-up for atypical intraductal hyperplasia and intraductal hyperplasia of the breast. *Cancer*, **65,** 518–529.

THEILE M, HARTMANN S, SCHERTHAN H, ARNOLD W, DEPPERT W, FREGE R, GLAAB F, HAENSCH W AND SCHERNECK S. (1995). Suppression of tumorigenicity of breast cancer cells by transfer of human chromosome 17 does not require transferred BRCA1 and p53 genes. *Hum. Mol. Genet.*, **10,** 439–447.

THOR AD, MOORE DH, EDGERTON SM, KAWASAKI ES, REISHAUS E, LYNCH HT, MARCUS JN, SCHWARTZ L, CHEN L-C, MAYALL BH AND SMITH HS. (1992). Accumulation of p53 tumour suppressor gene protein: an independent marker of poor prognosis in breast cancer. *J. Natl Cancer Inst.*, **84,** 845–855.

VAN DE VIJVER MJ, PETERSE JL, MOOI WJ, WISMAN P, LOMANS J, DALESIO O AND NUSSE R. (1988). *neu* protein over-expression in breast cancer: association with comedo- type ductal carcinoma *in situ* and limited prognostic value in stage II breast cancer. *N. Engl. J. Med.*, **319,** 1239–1245.

VARLEY JM, BRAMMAR WJ, LANE DP, SWALLOW JE, DOLAN C AND WALKER RA. (1991). Loss of chromosome 17p13 sequences and mutation of p53 in human breast carcinomas. *Oncogene*, **6,** 413–421.

VOJTESEK B, BARTEK J, MIDGELY CA AND LANE DP. (1992). An immunohistochemical analysis of human p53: new monoclonal antibodies and epitope mapping using recombinant p53. *J. Immunol. Methods*, **151,** 237–244.

WALKER RA, DEARING S, LANE DP AND VARLEY JM. (1991). Expression of p53 protein in infiltrating and *in situ* breast carcinomas. *J. Pathol.*, **165**, 203–211.

WINSHIP PR. (1989). An improved method for directly sequencing PCR-amplified material using dimethyl sulphoxide. *Nucleic Acids Res.*, **17**, 1266.

ZHUANG Z, MERINO MJ, CHUAQUI R, LIOTTA LA AND EMMERT-BUCK MR. (1995). Identical allelic loss on chromosome 11q13 in microdissected *in situ* and invasive human breast cancer. *Cancer Res.*, **55**, 467–471.